# Go beyond the limits of genetic algorithm in daily covariate selection practice

**DOI:** 10.1007/s10928-023-09875-7

**Published:** 2023-07-26

**Authors:** D. Ronchi, E. M. Tosca, R. Bartolucci, P. Magni

**Affiliations:** 1 Dipartimento di Ingegneria Industriale e dell’Informazione, Università degli Studi di Pavia, 27100 Pavia, Italy; 2 Clinical Pharmacology & Pharmacometrics, Janssen Research & Development, Beerse, Belgium

**Keywords:** Artificial intelligence, Machine learning, Genetic algorithm, Covariate selection, Automatic model building, Population PK/PD model

## Abstract

Covariate identification is an important step in the development of a population pharmacokinetic/pharmacodynamic model. Among the different available approaches, the stepwise covariate model (SCM) is the most used. *However, SCM is based on a local search strategy, in which the model-building process iteratively tests the addition or elimination of a single covariate at a time given all the others. This introduces a heuristic to limit the searching space and then the computational complexity, but, at the same time, can lead to a suboptimal solution.* The application of genetic algorithms (GAs) for covariate selection has been proposed as a possible solution to overcome these limitations. However, their actual use during model building is limited by the extremely high computational costs and convergence issues, both related to the number of models being tested. In this paper, we proposed a new GA for covariate selection to address these challenges. The GA was first developed on a simulated case study where the heuristics introduced to overcome the limitations affecting currently available GA approaches resulted able to limit the selection of redundant covariates, increase replicability of results and reduce convergence times. Then, we tested the proposed GA on a real-world problem related to remifentanil. It obtained good results both in terms of selected covariates and fitness optimization, outperforming the SCM.

**Supplementary Information:**

The online version contains supplementary material available at 10.1007/s10928-023-09875-7.

## Introduction

Pharmacokinetics (PK) and pharmacodynamics (PD) of a drug can significantly vary among individuals and/or groups of individuals. PK/PD modelling combined with a population (non-linear mixed effect—NLME) approach allows to account for the observed variability. The inclusion and quantification of individual-specific covariates increase the deterministic explanation of heterogeneity among individuals and facilitate its understanding. Therefore, covariate analysis is routinely performed during population PK/PD model building [1]. Accordingly, several algorithms for covariate selection are currently available [2–8]. Among them, stepwise approaches, in which covariates are added one-by-one, are the most widely used [9]. Although their application is widespread and entrenched in the pharmacometric field, this class of methods presents some non-negligible limitations [10]. Stepwise procedures are greedy algorithms which attempt to solve optimization problems by making locally optimal choices at each step. Consequently, they could return suboptimal solutions in some cases [9]. Additional problems could arise from the presence of moderate to high correlations between covariates and from the fact that the number of tested candidate covariates could affect the number of covariate included in the model [11].

Artificial intelligence (AI) and machine learning (ML) are strongly established in many research fields, and are becoming popular also in pharmacometrics [12–16]. AI/ML methods have been successfully adopted to support different tasks during the drug discovery and development process. For example, a plethora of quantitative structure–activity or property relationship (QSAR and QSPR) models based on ML has been proposed to predict physio-chemical characteristics as well as in vivo properties of candidate drugs [17–20]. In addition, AI/ML approaches were applied to identify complex associations and predict drug-drug interaction [21], or to design optimal strategies for precision dosing and medicine [22]. Among the successful applications of AI/ML to pharmacometric problems, the automatic model building and covariate selection is one of the most promising [23, 24].

Genetic algorithms (GAs) are ML methods inspired by the theory of natural selection [25], and they have been proposed as a valid alternative to the standard stepwise approaches for both automatic model building and covariate selection [16, 26–28]. The strength of the GA is that it is a global search method able to explore the entire search space of the candidate models, which represents all the hypotheses to be tested (e.g., number of compartments, covariates, random effects). However, the actual application of GAs during the PK/PD model building process is yet limited due to the extremely high computational costs and some convergence issues.

In this paper, we proposed a new GA for covariate selection to cope with and overcome some challenges associated with the current available algorithms [16, 26–30]. In particular, several strategies specifically designed to reduce computational times, improve convergence and limit the selection of highly correlated covariates were implemented. The proposed GA was applied on a simulated case study and on a real-world one related to remifentanil [31], demonstrating better performances compared to both previous implementations of GA and stepwise covariate modelling (SCM) as implemented in Perl-speaks-NONMEM (PsN).

## Theoretical

### Genetic algorithm

GA is an iterative and heuristic solution-search, inspired to evolutionary processes, to identify solutions to a given optimization problem [32]**.** GA is based on the metaphor of the Darwinian theory of evolution: an individual within a population must adapt itself to the environment to survive; similarly, a solution within a set of possible solutions must be adapted to solve the optimization problem to which it is associated [33]. The terminology used to describe the characteristics of genetic algorithms draws parallels with biology. The set of possible solutions is referred to as *population* and its size, which is defined beforehand, is referred to as *population size*. Each possible solution in the population, called *individual*, is represented by a unique codification, called *chromosome*, that summarizes all the characteristics of the individual. A chromosome is composed by *genes*, each of which codes for a specific somatic trait. The term *genotype* refers to the particular set of genes that characterize an individual and it is closely related to the concept of *phenotype*, which represents the corresponding set of somatic features. Each iteration of a GA is called *generation*. At each generation, only a subset of the population manages to survive and reproduce, creating new individuals by randomly recombining and mutating their genes. In this process, new features can emerge from the population, that prove to be beneficial for the survival of the individuals. These advantageous characters will therefore tend to be preserved and passed on to the offspring. After many generations, the population will evolve towards better individuals, which will be characterized by a higher *fitness*, i.e. a higher ability to adapt to the environment [32]**.**

### Genetic algorithm for covariate selection in population PK/PD modeling

In the context of covariate selection for population PK/PD modelling, the solution space of a GA is defined as the set of all possible PK/PD models with a different covariate structure. Each chromosome represents a candidate covariate model, and a population of chromosomes (set of models) is evaluated at each generation. The algorithm iteratively combines different covariate structures to find which covariate model better describe the data, trying to find the best trade-off between describing the data and limiting the number of selected covariates. The use of GA for covariate selection in population PK/PD modelling was first proposed by Bies et al. in 2006 [29], whose work become a reference in the field. Its application was further investigated to perform both covariate selection and automatic full model building in several subsequently works [16, 26–28, 30, 34], many of which from the same group.

GA showed good performances when compared to other automatic model selection algorithms in all the tested case studies. However, up to now its use in the daily work is still limited due to some relevant disadvantages. Although GAs are able to find a very good solution (local optimum), it cannot guarantee to identify the best one (global optimum) in a reasonable time due to the extent of the search space. This limitation could be outdated by increasing the population size to explore a broader portion of the search space. However, increasing the number of chromosomes in the population leads to an increase of the already high computational load that represents another relevant disadvantage of GA. Indeed, at each generation for each chromosome of the population the corresponding model has to be estimated on a given dataset to compute the value of the fitness function, which could require a lot of time. In this work, several strategies to address these challenges have proposed and successfully implemented.

## Methods

Starting from the algorithm proposed by Bies et al. [29], we developed a new GA specifically designed for covariate selection. The elements composing our GA as well as the implemented strategies are described in the following. Briefly, a configuration file, has to be defined by the user, then, the GA can be executed. First of all, the algorithm creates an initial population of chromosomes coding for the candidate covariate models (generation 0). Chromosomes are encoded with a suitable NM-TRAN script file of NONMEM and estimated on a given dataset through PsN. The outputs of the identification process are used to compute the value of the *fitness* function. Accounting for fitness values, a new generation of chromosomes is created applying a set of genetic operators. The process is repeated until the stop criteria are satisfied. A flowchart summarizing the steps of the proposed GA is reported in Fig. S1 of Supplementary Materials S1.

Implementation details are reported in Supplementary S1. In addition, GA scripts are available and can be freely downloaded for academic research purposes at http://aimed11.unipv.it/GAscript/.

### Elements of the genetic algorithm

#### The chromosome

Chromosomes, representing the possible covariate models, are coded by a binary representation. Every chromosome is comprised of several genes, each identifying a specific parameter-covariate pair. The length of each gene is given by ${{l}_{\mathit{gene}}=\mathrm{ log}}_{2}N$ (rounded up to the higher integer) where *N* is the number of parameter-covariate relationships being tested. As in the SCM function of PsN, for continuous covariates the linear, exponential, piece-wise linear and power relationships were considered. Differently for, for categorical covariates the options are not included or linearly included with an extra parameter added for each but the most common category. Therefore, genes coding for categorical covariates are always composed by 1 bit (0 = “no relationship”, 1 = “linear relationship”). Instead, the bits composing genes for continuous covariates range from 1 to 3 depending on the number of possible covariate models that the user wants asks to test through the configuration file: {0, 1} for two types of parameter-covariate relationship, {00, 01, 10} for three types, {00, 01, 10, 11} for four types and {000, 001, 010, 011, 100} for five.

**Fig. 1:**
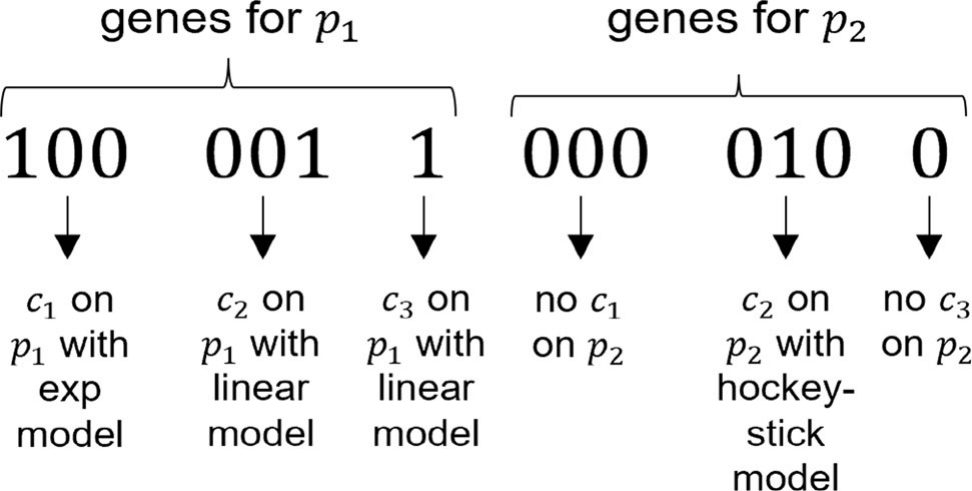
Example of a chromosome coding for a covariate model in which 2 continuous and 1 categorical covariates are included on first parameter and 1 continuous covariate on the second parameter

The length of the chromosome *l*_*chr*_ depends on the length of the genes and on the number of covariate-parameter pairs to be tested. For example, let’s consider having two parameters, p_1_ and p_2,_ two continuous covariates, c_1_ and c_2,_ and a categorical covariate, c_3,_ to test on both p_1_ and p_2_ with all the possible relationships_._ Thus, a possible chromosome for a candidate solution is reported in Fig. 1, and *l*_*chr*_ is 14 bits. The specific combination of the 14 bits is the genotype.

#### Genotype–phenotype mapping and NM-TRAN control stream creation

As exemplified in Fig. 1, each chromosome is decoded in a set of specific covariate-parameter relationships, the phenotype. The genotype–phenotype mapping (i.e., the translation of genes composing a chromosome into the corresponding covariate-parameter relationships) is not defined by default, but it depends on the instructions given by the user through the configuration file.

The decoded chromosome is then translated into a NM-TRAN *control stream* file of NONMEM that includes the base model and the covariate model. Based on information containing in the configuration file where the user specifies the covariate-parameter relationship to consider, the implemented workflow automatically writes the file. For each parameter of interest, first the algorithm considers the covariates to be test, distinguishing between continuous and discrete covariates, as different types of relationships are available for the two cases. Then, the effect of multiple covariates on a single parameter is implemented through a multiplicative model.

#### The model fitness function

In order to avoid overfitting, the GA search has to be guided by the trade-off between the goodness of data fit and the parsimony of model parameters. In particular, highly correlated covariates on the same model parameter have to be avoided. Accordingly, the fitness function is composed by three components:

- the objective function value (*obj*) resulting from model identification that represents the ability of the model to describe the data;
- a penalty term proportional to the number of covariates, *N*, included in the model;
- a penalty term, *ic*, that summarizes the correlations between continuous covariates on the same parameter and that is defined as
  $$ic=\frac{1}{2}\sum_{k=1}^{{n}_{par}}\sum_{i\ne j=1}^{{n}_{cov,k}}cor{r}_{i,j}$$

where *n*_*par*_ is the number of parameters affected by a covariate, *n*_*cov, k*_ the number of covariates included on parameter k and *corr*_*i,j*_ the correlation between* i*-th and *j*-th continuous covariates included on parameter *k*$.$

The fitness of the *c*th chromosome is, thus, defined as:

$$fi{t}_{c}= -\left(ob{j}_{c}+3.84*\left({N}_{c}+i{c}_{c}\right)\right)$$

where *N*_*c*_ represent the number of parameters required by the covariate relationships included in the *c*th chromosome and the weight 3.84 is selected because it represents the value of Chi Square distribution for a significance level of 0.05 and one degree of freedom.

#### Genetic operators

The algorithm is guided towards a solution to the given problem through the application of a set of genetic operators: selection, cross-over, mutation and elitism. The operators act stochastically, i.e., each is applied with a given probability.

##### Selection

This operator selects the individuals within a population which will form the mating pool for the reproductive process. Chromosomes with a higher fitness value have a higher probability to be selected to generate the new offspring, therefore focusing the research on promising regions of the search space. In this work, the *tournament selection* [35], characterized by the selection pressure parameter ${p}_{s}\in [\mathrm{0,1}],$ has been chosen. According to this selection strategy, first, two individuals are randomly sampled with re-entry within the population. Then, the two chosen chromosomes participate in a *tournament*: a random number between 0 and 1 is generated; if the value is lower than *p*_*s*_ the chromosome with the greatest fitness wins, otherwise, the other one is selected. The winner becomes one of the two parents which will create two offspring for the next generation. The process is repeated ${n}_{chr} /2$ times (where ${n}_{chr}$ is the number of chromosomes in the initial population), so that the new population will have the same size of the previous one. This type of operator, unlike other methods, does not restrict the possibility of recombination to only chromosomes with a high fitness. This is advantageous especially when the variance of the fitness is very high, to avoid losing potential well performing solutions from the combinations of individuals with a low fitness.

##### Crossover

To generate the new offspring, the chromosomes in the mating pool undergo potential recombination processes with probability *p*_*c*_. A single-point crossover has been applied: the two parent chromosomes are cut in a single randomly selected position and the two resulting parts of the parents are exchanged to form the new individuals.

##### Mutation

Once an offspring has been created, it undergoes the mutation process in which, with very low probability *p*_*m*_, each bit of the chromosome could switch (single-bit inversion mutation). The mutation operator represents the main variation tool.

##### Elitism

Through the elitism process a given percentage (% e) of the best individuals in the population of the *i*th-generation is copied and inserted directly into the (*i* +1)^th^ generation. This strategy ensures that the best chromosomes are not lost throughout generations.

### Codification issues after recombination and mutation processes

Crossover and mutation can produce chromosomes with genes that do not encode for any type of covariate-parameter relationship. To overcome this problem, the non-coding genes are replaced by the genes with the highest frequency in the best chromosomes of the prior generation.

### Initial population

**Fig. 2:**
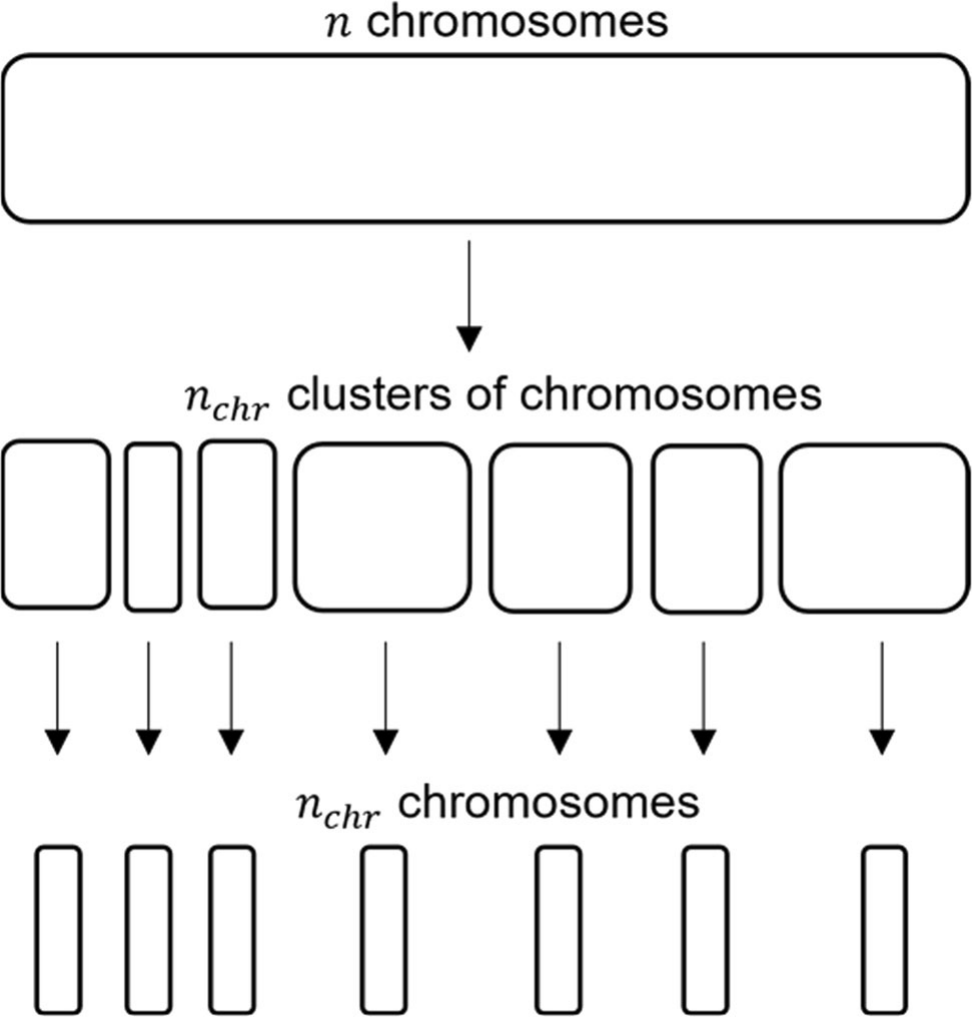
Schematic representation of the hierarchical clustering process to generate the initial population

In this step, the initial population of candidate solutions is generated. Initialization is generally based on a random generation of chromosomes. However, this may limit for chance the search space explored by the GA and it may affect the convergence performance, especially when the population size is small with respect the search space. To overcome this issue, in this work, a novel strategy based on hierarchical clustering [36] has been proposed to maximize the heterogeneity between the chromosomes of the initial population, therefore maximizing the portion of the represented search space from the beginning, while keeping the population size relatively small. The steps to generate the initial population, represented in Fig. 2, are:

- randomly generate $n$=20*${n}_{chr}$ chromosomes, where ${n}_{chr}$ is the desired population size;
- calculate the matrix of the distance between chromosomes. The Jaccard distance, *Jd*, is used. Given a pair of binary vectors (*i*, *j*), *Jd* is defined as:
  $$Jd\left(i,j\right)=\frac{b+c}{a+b+c}$$

where $a$ is the number of bits equal to 1 in both $i$ and $j, b$ is the number of bits equal to 0 in object $i$ and to 1 in object $j$, and $c$ is the number of bits equal to 1 in object $i$ and to 0 in object $j$;

- perform the hierarchical clustering on the basis of that distance matrix;
- cut the dendrogram at ${n}_{chr}$ clusters;
- randomly select one chromosome from each cluster;
- pool the ${n}_{chr}$ selected chromosomes to form the initial population.

In Supplementary S2, an example of random and cluster-based initial population distribution is reported as proof of concept.

#### Stopping criteria and optimal solution selection

GA stops automatically when it reaches the pre-defined number of generations (*d*). Then, the model with the maximum fitness value among all the chromosomes that has been assessed during the search is selected as optimal model. Choosing the appropriate value of *d* can be challenging and depends on the complexity of the problem. Therefore, a log-file reporting aggregate statistics of fitness values, i.e., mean, range, variance, and standard deviation, is available for monitoring purpose and updated at the end of each generation. Monitoring the convergence dynamics reported in the log file, at any time the user can choose to stop or to continue the GA, also extending the number of generations.

### Improving GA efficacy: heuristics for time-issues

The computational cost is one of the major limitations of GAs when applied to covariate selection or automatic model building. In this work, three strategies have been adopted to speed-up the execution. First of all, the algorithm implementation allows the parallelization of the chromosome processing in an arbitrary number of cores. This design strategy is fundamental to leverage cloud/cluster computing services or multi core architectures and take full advantage of the computational power of the selected infrastructure.

Further, preliminary results showed that, during the search, the GA also tested covariate models with a very complex structure that required a lot of time to be estimated, also because the increased number of parameters. Studying the correlation between the length of the model execution and the optimality of the covariate structure, we observed that models with the longest executions times generally performed poorly, i.e., were characterize by low value of fitness function. Therefore, the GA could be significantly slowed down by executing models that are far from the optimal solution. To overcome this issue, it was decided to stop models with longer estimation times. The *timeout* command-line utility in Linux was used to this scope. Starting from the second generation, all the models for which the estimation process is longer than a threshold *t*_*th*_ are forcefully terminated, and a very low fitness value is assigned to the corresponding chromosomes. *t*_*th*_ is set equal to the 95th percentile of the execution times of the models that have successfully converged in the previous generations and is updated at each generation.

Finally, the log-file containing information about the composition of all generated chromosomes (updated at the end of each generation) is used as a lookup table. The file is consulted whenever a new chromosome is generated: if the corresponding model has been already run, the estimation process is not repeated, and the previously computed fitness value is simply reused.

### Evaluation of the new GA on two case studies

The proposed GA was first developed and tested on a simulated case study. First, a data rich scenario was used to assess all the new design options introduced in the algorithm. In particular, the effects of including the ${ic}_{c}$ penalty in the fitness function, of the clustering population initialization and of the timeout utility were evaluated. To this aim, four different implementations of GA designed with an incremental approach were considered. Starting from a base version of the algorithm (*Base GA*) that mimicked the GA originally proposed in the literature [26, 28], the ${\mathrm{ic}}_{\mathrm{c}}$ penalty, clustering initialization and timeout utility were included incrementally. The different design options were evaluated in terms of (i) optimality of selected covariate model compared to the “true model” used to generate the simulated data, (ii) robustness of converging to the same optimal solution and (iii) computational efficiency.

Then, the performances of the *Complete GA*, including the ${\mathrm{ic}}_{\mathrm{c}}$ penalty, clustering initialization and timeout utility, were further assessed on several simulated scenarios characterized by different data richness, to evaluate the robustness of the results with respect to different generated datasets and more realistic sparce sample scenarios.

Finally, the *Complete GA* was tested on a real-world case study related to the remifentanil [31], and its performances were compared with those of the SCM as implemented in PsN.

#### Simulated case study

The same simulated case study used by the Bies’s group [26] was considered. It consisted of a 1-compartment model with linear elimination, describing the pharmacokinetics of a generic drug after a single intravenous (iv) administration. The model was parameterized in terms of volume of distribution V, and clearance CL. Log-normally distributed inter-individual variability was assumed on both the parameters. The covariate effects added to the parameters were creatinine clearance (CRCL) and body mass index (BMI) with and exponential equation on V, and body surface area (BSA) and sex (SEX) on CL with exponential and linear model, respectively.

Simulated individuals were characterized by the set of covariates included in the “true model”, i.e., BMI, BSA, CRCL and SEX, and by a set of confounding covariates (spurious), i.e., age (AGE), creatinine (CR), height (HT) and weight (WT). First, SEX was sampled from a discrete uniform distribution (0,1). Then, BMI, AGE, CR, HT were supposed to be uncorrelated given the SEX and were sampled from normal (AGE) and lognormal distributions (BMI, CR, HT). In particular, the mean of HT distribution differed for male and female. Finally, the dependent covariates, i.e., WT, BSA, CRCL, were computed as function of the uncorrelated covariates: WT = *BMI*·*HT*^2^ , BSA = $\sqrt{\left(HT\cdot 100\cdot WT/3600\right)}$ and CRCL = 140·*AGE*·*WT*/(72 * *CR*). Parameters of the covariate distributions together with details about the simulated virtual population are reported in the Supplementary Material S3.

Drug concentration values were simulated at 0.25, 0.5, 1, 2, 3, 6, 10 and 24 h after dose assuming a combined residual error model. Model parameter values used for simulation are reported in the Supplementary Material S3.

To consider the entire search space, all the covariates were possible tested on each parameter considering all the possible relationship types, as specified in the configuration file reported in Supplementary S3. The hyper-parameters of the algorithm were set to the values reported in Table 1.

**Table 1:** Value of the hyper-parameters of the GA used for the simulated and the remifentanil case-study

Hyper-parameter	Definition	Simulated case study	Remifentanil case study
*p*_*s*_	Selection pressure parameter	0.75	0.75
*p*_*c*_	Crossover probability	0.7	0.7
*p*_*m*_	Mutation probability	0.025	0.025
% e	Percentage of chromosomes preserved by elitism	10%	10%
*n*_*chr*_	Number of chromosomes in the population	30	50
#*Gen*	Number of generations	50	150

#### Remifentanil case study

Remifentanil is an opioid analgesic drug with a rapid onset and recovery time; it is used for sedation and, in combination with other drugs, for general anaesthesia. Remifentanil is generally administered through an iv infusion at a constant rate between 1 and 8 μg kg^−1^ min^−1^ for 4–20 min. Its PK had been previously assessed in 65 healthy adults [31]. For each patient, several covariates were available in the dataset: five continuous (AGE, HT, WT, BSA, Lean Body Mass—LBM) and 1 categorical (SEX). The PK was described by a 3-compartment model with lognormal inter-individual variability on all the parameters (CL, V1, Q2, V2, Q3, V3) and a proportional residual error.

As already done for the simulated case study, to consider the entire space of solutions, all the candidate covariates were tested on each model parameter considering all the possible relationships, as detailed in the configuration file reported in Supplementary S4. GA was applied using all the strategies described in the methods section, including the clustering population initialization and the timeout strategy. The hyper-parameters of the algorithm used for this case-study are reported in Table 1.

The same covariate search was performed with the SCM function of PsN considering both the search directions and p-value threshold equal to 0.05 and 0.01 for forward and backward direction, respectively.

## Results

### Simulated case study

A rich dataset including 200 subjects and 8 samples for subject was simulated and used to evaluate the new proposed heuristic introduced in the GA. To assess the robustness of the algorithm among several runs, each version of the GA was executed 20 times and the best selected covariate model (i.e., that with the lowest *fitness* value) across the 20 runs was considered. All the GA versions identified the same optimal model, reported in Table 2, at least once. Model parameter estimates are shown in Table 3 and compared with the ones of the base model (without covariates) and of the true model re-estimated on the simulated dataset.

**Table 2:** Covariate model structure of the true model and of the optimal solution selected by the GA

Model parameter	Covariates	
	True model	GA solution
CL	CRCL-exponential	CRCL- piece-wise linear
	BMI-exponential	BMI-exponential
V	BSA-exponential	SEX-linear
	SEX-linear

**Table 3:** Parameter estimates for the base model (without covariates), the true model and the model selected by the GAs

Parameter	Base model (without covariates)	True model (estimated on the simulated dataset)	GA–solution
CL	0.654	0.608	0.528
V	1.94	2.28	2.34
BMI on CL (exponential)	–	0.224	0.232
CRCL on CL (exponential)	–	0.272	–
CRCL on CL (piece-wise linear–1)	–	–	0.0162
CRCL on CL (piece-wise linear–2)	–	–	0.543
BSA on V	–	0.0676	–
SEX on V	–	− 0.347	− 0.32
CL—inter-individual variability (random effect)	0.371	0.2	0.196
V—inter-individual variability (random effect)	0.299	0.25	0.256
Correlation between CL and V	0.0766	0.0497	0.048
Additive error	0.10	0.106	0.106
Proportional error	0.000365	0.00036	0.000361
OBJ	− 6388.597	− 6528.256	− 6531.310
AIC	− 6384.597	− 6516.256	− 6519.310

The GAs correctly selected three of the four covariates, i.e., CRCL and BMI on CL and SEX on V, but missed to identify the effect of BSA on V. In addition, the effect of CRCL on CL was modelled by a piece-wise linear relationship instead of an exponential one. No spurious covariates were included in the model, despite the high correlations between candidate covariates. Even if the solution identified by the GAs was slightly different from the true covariate model it resulted better than the true model in terms of objective function value (− 6528.256 vs − 6531.31) and AIC (− 6516.256 vs − 6519.31). In addition, as it can be noticed from Table 3, parameter estimates of the true model and of GA suggested model were in good agreement. The estimated inter-individual variability on CL and V (random effect) was comparable. To investigate why the BSA was not automatically included on V by the GAs, it was manually added to the selected model with an exponential relationship and the model re-estimated. A non-significant improvement in the AIC (− 6519.78 vs − 6519.31) was obtain, suggesting that the effect of the true covariate could be too weak to be detected in this single simulated scenario.

Once the best solution across the 20 runs had been assessed, we considered the robustness of the different GA implementations of converging to it. As reported in Table 4, the *Base GA* selected the best solution in 55% of the runs, while in the 45% of the cases it converged to more complex models characterized by more spurious covariates and a lower AIC. Each of the new strategies introduced in the algorithm increased the probability of GA to converge to the best solution. The percentage of runs selecting the optimal model raised from 55 to 65% introducing the *ic*_*c*_ penalty in the fitness function, further increased to 80% due to the clustering population initialization and, finally, reached 95% (19/20) when also the timeout utility was include (*Complete GA*).

**Table 4:** Summary statistics on the 20 runs of the GA in the different conditions

	Base GA	Updated GA—version 1	Updated GA—version 2	Complete GA
Correlation penalty	x		x	x
Clustering initialization			x	x
Timeout utility				x
Runs selecting the best solution	55%	65%	80%	95%
Mean execution time for 50 generations	63.6 min	62.8 min	62.5 min	60.8 min
Mean number of generations needed to reach the optimal solution	32.75	31.23	26.75	27.15
Mean execution time to reach the optimal solution	45.2 min	43.9 min	38.5 min	37.2 min

Regarding the computational time, no significant differences were observed in terms of mean execution time to perform 50 generations among the different approaches. However, the introduction of clustering population initialization significantly reduced the number of generations needed to reach the optimal solution (26.75 vs 32.75). Resulting in a 12.9% reduction of the mean execution time needed to reach the best solution. The timeout strategy gave an additional reduction of − 2.9% in computational time.

**Fig. 3:**
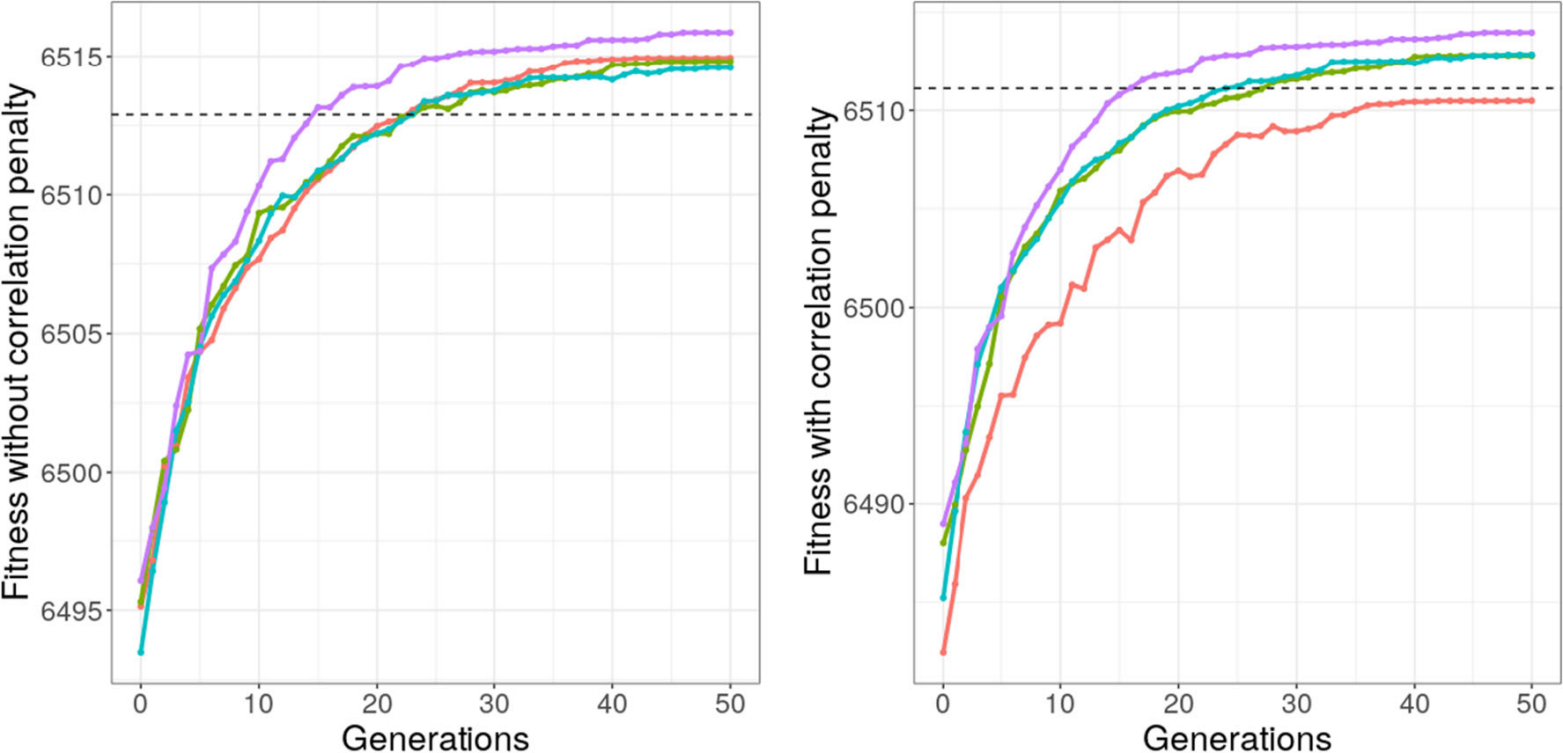
Evolution of the average maximum fitness over generations (over 20 runs of the algorithm). In violet the Complete GA, in light-blue the version 2 of the GA, in green the version 1 of the GA and in red the Base GA. In black the true model (Color figure online)

Finally, the evolution of the average maximum fitness value across different runs is reported in Fig. 3. Since the fitness function of the new proposed versions of GA differed from the one of the Base GA, due to the introduction of the *ic*_*c*_ penalty, results were compared considering both the fitness function definitions. It is worth noting that in both the cases the Complete GA outperformed the Base GA.

In summary, the *Complete GA*, due to the three novel heuristics here introduced, outperformed the *Base GA,* inspired to literature algorithms [28], demonstrating higher performances in robustly and efficiently converging to a better solution.

After the assessment of the improvement provided by the new design options, we evaluated the robustness of the *Complete GA* performances with respect to (random) dataset generation and the data richness (sample per subject). To this end, first we generated 20 replicates of the rich simulated dataset (200 subjects, 8 samples per subject). Then, we considered two additional scenarios with sparser data, sub-sampling 5 (0.25, 0.5, 2, 6, 24 h) or 3 (0.25, 2, 6 h) samples per subject from the rich datasets. For each replicate and scenario, the *Complete GA* was run, and results summarized across the replicates of the dataset (see Table 5).

**Table 5:** Summary statistics about covariate model selected by the Complete GA on 20 replicates of three simulated scenarios

	8 samples/id	5 samples/id	3 samples/id
True covariates*			
Runs including CRCL on CL	85%	75%	80%
Runs including BMI on CL	95%	95%	95%
Runs including BSA on V	40%	45%	55%
Runs including SEX on V	100%	100%	85%
Average number of included covariates [25th p, 75th p]	3.2/4 [3, 4]	3.15/4 [3, 4]	3.15/4 [3, 4]
*Spurious covariates*			
Average number of included covariates [25th p, 75th p]	1.3 [1, 2]	1.25 [0, 2]	1.2 [0, 2]
*Covariate model optimality*			
Number of cases where *AIC*_*GA solution*_ ≤ *AIC*_*true model*_	18/20	17/20	19/20
*Computational efficiency*			
Mean execution time for 50 generations (CV%)	54.8 min (5.2%)	53.9 min (4.4%)	53.4 min (4.2%)
Mean number of generations needed to reach the optimal solution (CV%)	28.57 (37.4%)	27.7 (31.4%)	30.1 (38.3%)
Mean execution time to reach the optimal solution (CV%)	37.6 min (28.4%)	32.1 min (28.5%)	33.51 min (32.9%)

*Unrespect to the type of covariate-parameter relationship

First, we evaluated the covariate selected by the GA compared to the true model. As reported in Table 5, the *Complete GA* was able to consistently identify the true covariate structure across the replicates of the dataset and unrespect to data richness. Indeed, for the rich data scenario the GA solution always included at least three of the four true covariates and only few spurious covariates (1.3 in average). In agreement with previous results, in almost all the replicates of the dataset the GA correctly included the CRCL and BMI on CL and SEX on V. Differently, the BSA-V relationship was more challenging to be identified, however, it was correctly selected in the 40% (8/20) of the replicates. Even when slightly different from the true covariate model, the GA solution was generally better than the true model in terms of AIC. This finding is not surprising. Finally, equivalent results were obtained for the two scenarios characterized by sparser data (5 and 3 samples per subject), demonstrating that the *Complete GA* was robust also respect to the data richness.

In addition, the GA performances relating to the number of generations needed to reach the optimal solution and computational time required were not significantly affected.

More detailed results are provided in the Supplementary S5.

### Remifentanil case study

The complete version of the GA proposed in this paper was then applied on the remifentanil case study and compared to the SCM. To allow comparison with GA results, we asked the SCM to test all the candidate covariates on each model parameter considering all the possible relationships. The choice of testing a range of model alternatives (i.e., linear, hockey-stick, exponential and power models) for each continuous covariate introduced a dependence of the SCM results on the order in which the parameter-covariate relationships were tested. Therefore, the SCM was executed multiple times changing in a combinatorial way the order in which the relationships for each covariate-parameter pair were tested to ensure that SCM results were not biased by the starting points and to increase the likelihood of finding the global optimum. To increase comparability of the results, also the GA was executed 10 times. The overall best solution (in term of AIC) identified by the GA and SCM is reported in Table 6. The two covariate models were very similar, with differences only for the covariates included on CL (WT by GA and BSA by SCM) and on V3 (HT, AGE and SEX by GA and LBM by SCM).

**Table 6:** Covariate model structure of the overall best solution selected by GA and SCM

Model parameter	Covariates	
	scm solution	Complete GA solution
CL	AGE—exponential	AGE—exponential
	BSA—exponential	WT—exponential
V1	LBM—exponential	LBM—exponential
Q2	AGE—exponential	AGE—exponential
V2	AGE—exponential	AGE—exponential
	SEX—linear	SEX—linear
Q3	AGE—piece-wise linear	AGE—piece-wise linear
V3	LBM—exponential	AGE—exponential
		HT—piece-wise linear SEX—linear

**Fig. 4:**
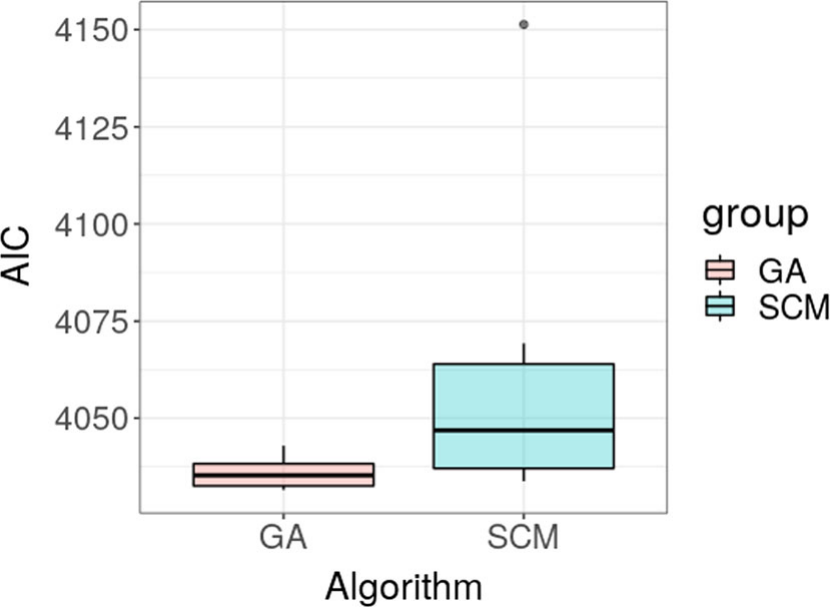
Boxplot of the AIC score for the model selected by different runs of GA (Pink box) and SCM algorithm (Blue box) (Color figure online)

Comparing the AIC value, the best model selected by the GA was slightly better (slightly lower AIC) than the best solution identified by the SCM (4031.48 vs 4033.74) among the 24 runs. However, the solution provided by the SCM approach was relevantly influenced by the order of the shape of relationships in which the candidate covariates were tested. Indeed, considering all the 24 SCM runs, the AIC of the selected models ranged from 4033.74 to 4151.23. On the other hand, even if also the GA solution varied among different runs due to the stochasticity of the evolutionary process, the AIC range of the models selected by the GA was narrower, from 4031.48 to 4042.9 (Fig. 4). The structure of the models selected by the GA in each run are reported in Supplementary S4.

## Discussion

GA has been suggested as a useful tool for automatic covariate selection in population PK/PD modelling, and good performances have already been obtained [16, 26, 27, 34]. However, some relevant limitations prevent its actual use in the daily pharmacometric modelling activity. In this work, a new GA for covariate selection has been proposed. The structure of the developed algorithm was inspired by the GAs already available in the literature for model building and covariate selection (*Base* GA). However, new strategies were proposed to overcome issues encountered in the previous works, with particular focus on the risk of falling in a local minimum, the high computational load, and the tendency of including highly correlated covariates.

The size of the chromosome population is an important hyper-parameter of GA, and it can significantly affect GA performances and efficiency. Indeed, if the population dimension is too limited, GA would explore a reduced portion of the search space, with consequent increasing in the risk of falling into a local minimum. Otherwise, increasing the population size leads to a higher computational load. In this work, a strategy based on hierarchical clustering has been proposed to maximise the heterogeneity of the initial population and, thus, widen the region of the search space explored by the GA while maintaining the same number of chromosomes. The advantages of the clustering approach were demonstrated by comparing the result of the algorithm with and without initialization strategy. The non-random population initialization improved the ability of the GA to find the best solution (see Table 4).

With regard to computational load, GA was allowed to process chromosome in parallel with an arbitrary number of cores. In addition, a timeout utility has been implemented to early stop the run of non-promising candidate models, using prior information on the execution time that a model generally required to be estimated for the considered problem in a given hardware architecture. Indeed, it has been observed that the most promising chromosomes are associated with a lower execution time. When applied to the simulated scenario, the introduction of the timeout strategy did not significantly decrease the computational time. However, it significantly improved the GA convergence performances, allowing to select the best solution in 95% of the runs instead of 80% (as shown in Table 4). The lack of improvement in the execution time likely followed by the fact that, due to the simplicity of the considered case study (linear one-compartment model), the difference between the execution times of a good and a bad model was narrow. When applied to more complex scenarios, the timeout strategy is expected to have greater impact on running times. As proof of concept, 10 generations of the GA-version 2 (without the timeout utility) are performed on the remifentanil case study and compared with 10 generations of the complete GA. It is observed that the time needed by the GA-version 2 is 19.7% longer than the time required by the complete version of the algorithm (542.9 vs 649.9 min). The observed 19.7% increase of computation time is much larger than the one observed in the simulated case study where the GA-version 2 need only 2.9% longer time than the time required by the complete GA in performing 50 generations (62.5 vs 60.8 min). The effect of the timeout utility is then surely made less pronounced by the parallel execution of several chromosomes, indeed, even without the timeout strategy, the algorithm can proceed despite some cores being stacked in solving an unpromising chromosome.

To avoid the introduction of highly correlated covariates on the same parameter, a penalty coefficient proportional to the covariate correlation was introduced in the definition of the fitness function. This penalty had a significant impact on guiding the GA through the selection of simpler models. Indeed, when the correlation penalty was removed, in 45% of the runs GA selected sub-optimal models including several spurious covariates characterized by high correlations. The issue was already highlighted by Sherer and co-authors [26]. Indeed, in the same simulated case study here considered, the GA proposed by Sherer et al. identified a solution including three of the four true covariate relationship (i.e., CRCL and BMI on CL and SEX on V) and three spurious ones. To mitigate this problem the authors increased the point penalty per covariate included in the model and adding a user defined penalty for highly correlated parameters. The approach proposed in this work has the advantage to be user independent.

Finally, the complete version of the GA was tested on the real case study of remifentanil [31]. This example has been used, among several others, in the work of M. Prague, M. Lavielle [7]*,* in which a new tool for automatic model building is proposed and compared with SCM and Conditional Sampling for Stepwise Approach (COSSAC), and in which the best solution was provided by SCM. In our work, both GA and SCM were run several times. On the one hand performing multiple runs allowed to account for the stochastic nature of GA and on the other it ensured that the SCM results were not affected by the initial conditions. Indeed, it was observed that the model selected by SCM was strongly affected by the order in which the covariate-parameter relationships were tested. This dependence derived from the specific choice of testing multiple model alternatives (i.e., linear, hockey-stick, exponential and power models) for each continuous covariate-parameter combination. Testing such a range of covariate-parameter relationships is not recommended in the every-day practice of covariate model building, thus, performing multiple runs of SCM is not necessary in the standard SCM usage. However, in this work we exceed the scope of the daily practice in covariate selection and challenged the GA and SCM to identify the optimal covariate model without the need of restricting the space of possible solutions by introducing a priori assumptions on the covariate-parameter combinations or the types of relationship to test. In this context, the GA outperformed the SCM according to the best selected model among different runs (AIC of 4031.48 vs 4033.74), and according to the AIC distribution of the selected models. Indeed, even if also the GA identified different solutions among the different runs, the selected models were almost equivalent both for structure and performance (AIC).

In summary, in this work a new GA for covariate selection has been proposed, further confirming that GA is an attractive approach, which could compete with more standard methods for covariate selection. When applied to a real case study, our GA proved to perform better than SCM by selecting models with greater fitting performances. In addition, the strategies proposed, i.e., the population initialization by hierarchical clustering to limit the risk of falling in local minimum, the parallel implementation and timeout utility to manage computational load, and the correlation penalty to avoid inclusion of high correlated spurious covariates, represented relevant improvements compared to GAs previously proposed.

Some additional considerations must be addressed regarding the computational load. The total runtime of the GA strongly depends on GA hyperparameters and model complexity. Although the execution time of the proposed GA required only one hour for the simulated case scenario (1 compartment PK model with linear elimination), when applied to the remifentanil case study its execution time reached approximately 100 h. This strong difference is an implicit consequence of the longer time required by NONMEM to identify more complex models and does not depend on GA convergence issues. Comparing the computational load of GA and SCM, it is evident that a single SCM run takes a significantly shorter time than GA (about 2.5 min for the simulated scenario and 2.5 h for the real one). However, given the high dependence of SCM solution from the order in which the parameter-covariate relationships are tested, SCM could require several runs to obtain a robust solution, reducing the computational advantage. Differently, multiple runs of GA returned more consistent solutions, suggesting the unnecessity of running GA several times. Despite the improvements proposed in this work, computational time required by GA remains high. However, the parallel implementation of GA would allow to exploit powerful computational architecture, such as cloud computing, grid computing or other shared resources methods, decreasing the run times. In addition, alternative strategies could be evaluated to further reduce the execution times. For example, the introduction of an hybrid component of the algorithm, that complements the globally-oriented genetic algorithm with a local search method [29], had been proposed in the literature, and accordingly to Sibieude et al. 2022 [34], it could give another − 34% benefit on the computational load.

## Conclusions

The appropriate inclusion and quantification of individual-specific covariates is a major task of the PK/PD model building exercise. Consequently, selection of covariates is routinely performed during pharmacometric analysis, and a number of algorithms are currently available. The GA is a ML method inspired by natural selection theory that has been recently proposed as an efficient and viable solution. Nevertheless, until now some limitations of the algorithm have limited its development in daily practice. In this paper a new GA for covariate selection has been proposed, providing further evidence that GA is an appealing approach, that could rival the more standard methods for covariate selection. In addition, the strategies proposed, i.e., the population initialization by hierarchical clustering to limit the risk of falling in local minimum, the parallel implementation and timeout utility to manage computational load, and the correlation penalty to avoid inclusion of high correlated spurious covariates, represented a relevant step toward more widespread use of GAs for covariate search.

### Supplementary Information

Below is the link to the electronic supplementary material.

Supplementary file1 (PDF 280 KB)

Supplementary file2 (PDF 120 KB)

Supplementary file3 (PDF 135 KB)

Supplementary file4 (PDF 94 KB)

Supplementary file5 (PDF 105 KB)
