# Supplementary material for: Go beyond the limits of genetic algorithm in daily covariate selection practice
Source: J Pharmacokinet Pharmacodyn. 2023 Jul 26;51(2):109–21. doi: 10.1007/s10928-023-09875-7 (PMC10982092; doi:10.1007/s10928-023-09875-7)
Supplement: Supplementary file 1 — Supplementary file1 (PDF 280 KB) [file 10928_2023_9875_MOESM1_ESM.pdf]

**Journal of Pharmacokinetics and Pharmacodynamics** (Original Paper)

**TITLE:**

# Go beyond the limits of Genetic Algorithm in daily covariate selection practice

**Authors:** D. Ronchi[1], E.M. Tosca[1], R. Bartolucci[1,2], P. Magni[1]

**Date:** Received: data/ Accepted: date

1. Dipartimento di Ingegneria Industriale e dell'Informazione, Università degli Studi di Pavia, 27100 Pavia, Italy
2. Clinical Pharmacology & Pharmacometrics, Janssen Research & Development, Beerse, Belgium

**Corresponding author:**

Paolo Magni paolo.magni@unipv.it

**Supplementary Section 1**

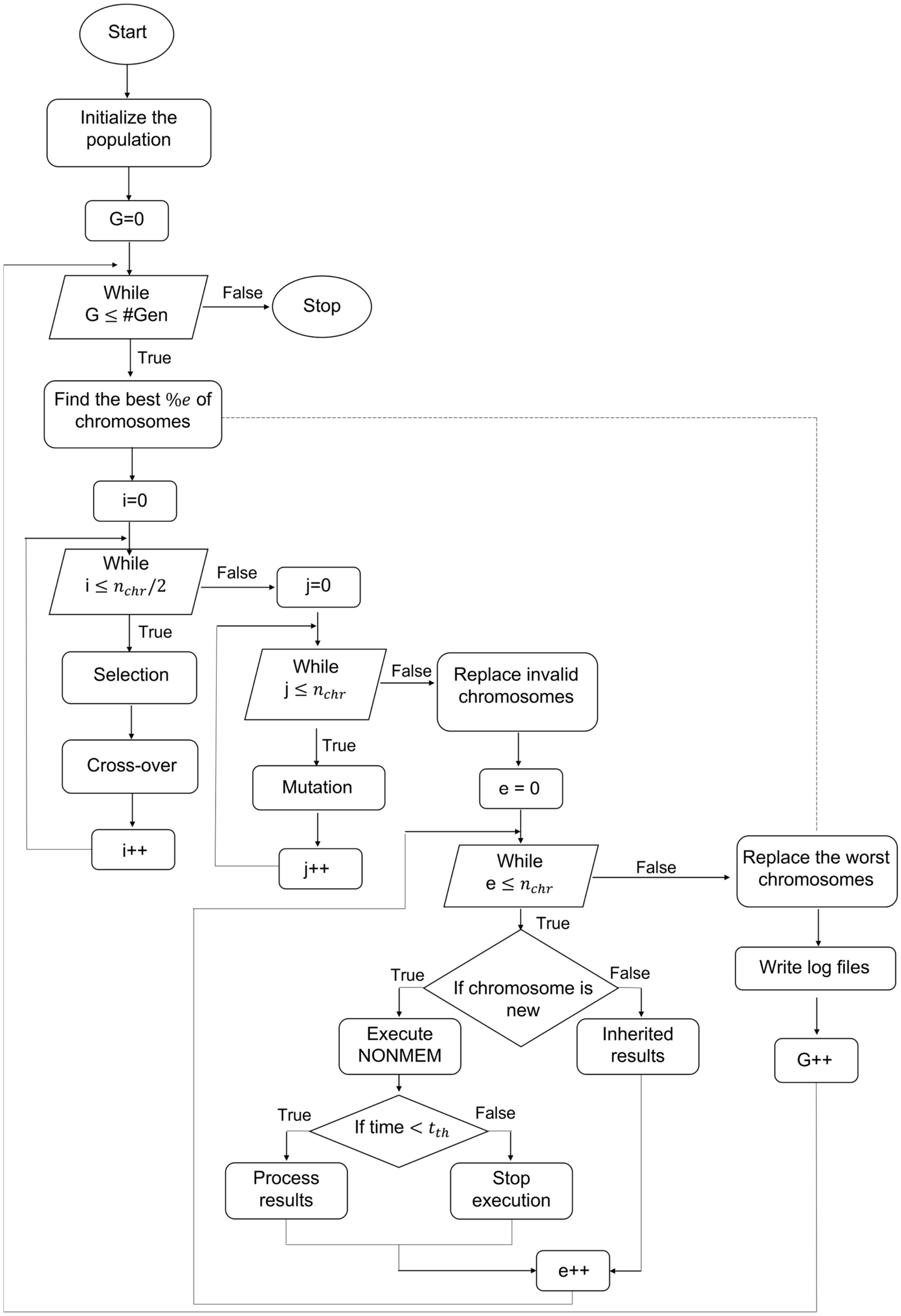


***Fig. S1*** *Flowchart of our GA for covariate selection.* $n_{chr}$ *is the population size,* $\#Gen$ *is the maximum number of generations,* $t_{th}$ *the time threshold.*

## Software

The GA is written in Perl (ver. 5.010). NLME models are implemented in NONMEM 7.4. The script for the generation of the initial population is written with R. The GA can be executed from terminal or inside RStudio environment. Performances were tested on an 8x Intel(R) Core (TM) i7-7700 CPU @ 3.60GHz machine with with Lubuntu 20.04.

GA scripts are available and can be freely downloaded for academic research purposes at http://aimed11.unipv.it/GAscript/.

## Configuration file

Before running the covariate selection GA, as preliminary step, a configuration file, similar to that of the PSN SCM function, has to be defined. It is composed by several sections:

- In the first section, the user must specify i) the name of the script file of the base model, ii) the continuous and categorical covariates to be tested, which have to be present in the data file
- In the *[test_relations]* section, the user must specify which combinations of parameter-covariate have to be tested.
- In the *[valid_state]* section, the user must assign the type of relationship for each parameter-covariate pair being tested, following this codification:
  - 1 – no relationship
  - 2 – linear relationship
  - 3 – piece-wise linear relationship
  - 4 – exponential relationship
  - 5 – power relationship.

For categorical covariates, only 1 and 2 can be investigated.

An example of configuration file is reported below.

```
;;;;; Start of the configuration file ;;;;;;

model=BaseModel.mod ; i.e. : 1-compartment-model

continuous_covariates=CovCont1, CovCont2, CovCont3

categorical_covariates=CovCat1, CovCat2

[test_relations]

CL= CovCont1, CovCont2, CovCont3, CovCat1, CovCat2

V= CovCont1, CovCont2, CovCont3, CovCat1, CovCat2

[valid_states]

continuous=1,2,3,4,5

categorical=1,2

;;;;; End of the configuration file ;;;;;;
```
