# Supplementary material for: Go beyond the limits of genetic algorithm in daily covariate selection practice
Source: J Pharmacokinet Pharmacodyn. 2023 Jul 26;51(2):109–21. doi: 10.1007/s10928-023-09875-7 (PMC10982092; doi:10.1007/s10928-023-09875-7)
Supplement: Supplementary file 2 — Supplementary file2 (PDF 120 KB) [file 10928_2023_9875_MOESM2_ESM.pdf]

Journal of Pharmacokinetics and Pharmacodynamics (Original Paper)

TITLE:

Go beyond the limits of Genetic Algorithm in daily covariate selection practice

Authors: D. Ronchi[1], E.M. Tosca[1], R. Bartolucci[1,2], P. Magni[1]

Date: Received: data/ Accepted: date

1. Dipartimento di Ingegneria Industriale e dell'Informazione, Università degli Studi di Pavia, 27100 Pavia, Italy
2. Clinical Pharmacology & Pharmacometrics, Janssen Research & Development, Beerse, Belgium

Corresponding author:

Paolo Magni paolo.magni@unipv.it

Supplementary Section 2

Projection in two dimensions with t-distributed stochastic neighbour embedding (t-SNE)[1] of population randomly generated and a population obtained by applying clustering initialization. The non-randomly initialized population has a lower spatial density because chromosomes are spaced further apart.

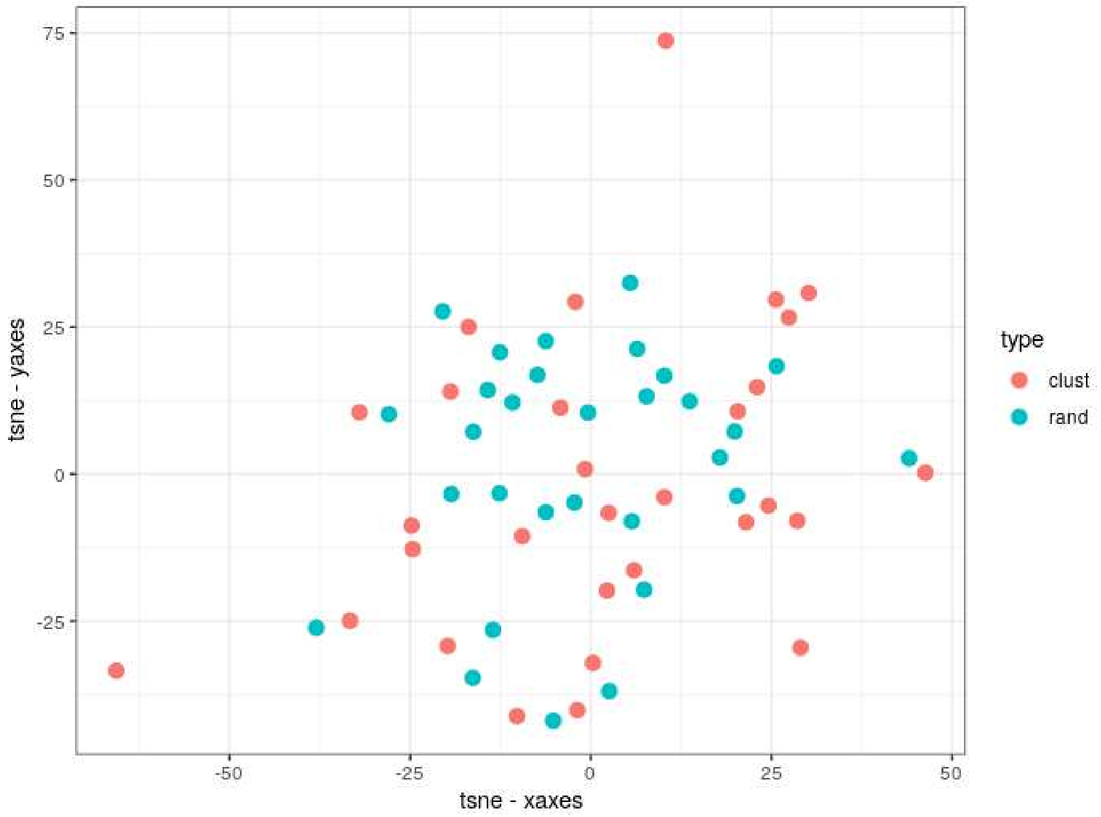

Fig. S2 *tSNE of chromosome in initial population*

1. Maaten, L. van der & Hinton, G. Visualizing Data using t-SNE. *J. Mach. Learn. Res.* **9**, 2579–2605 (2008).
