# Supplementary material for: Go beyond the limits of genetic algorithm in daily covariate selection practice
Source: J Pharmacokinet Pharmacodyn. 2023 Jul 26;51(2):109–21. doi: 10.1007/s10928-023-09875-7 (PMC10982092; doi:10.1007/s10928-023-09875-7)
Supplement: Supplementary file 3 — Supplementary file3 (PDF 135 KB) [file 10928_2023_9875_MOESM3_ESM.pdf]

Journal of Pharmacokinetics and Pharmacodynamics (Original Paper)

TITLE:

Go beyond the limits of Genetic Algorithm in daily covariate selection practice

Authors: D. Ronchi[1], E.M. Tosca[1], R. Bartolucci[1,2], P. Magni[1]

Date: Received: data/ Accepted: date

1. Dipartimento di Ingegneria Industriale e dell'Informazione, Università degli Studi di Pavia, 27100 Pavia, Italy
2. Clinical Pharmacology & Pharmacometrics, Janssen Research & Development, Beerse, Belgium

Corresponding author:

Paolo Magni paolo.magni@unipv.it

Section 4: Additional information for the simulated case study

Table S4.1: Typical values and standard deviation of covariates used to simulate the 200 patients

Covariate	Mean	Standard Deviation
BMI	26 kg/m^2	4 kg/m^2
HT	1.6m (1.5 m female – 1.7 m male)	0.2 m
CR	1.0 mg/DI	0.13 mg/DI
AGE	40 years	8 years
SEX	-	0.5 female – 0.5 male
WT	-	-
BSA	-	-
CRCL	-	-

Table S4.2: Typical values and standard deviation of the simulated population of 200 patients

Covariate	Mean	Standard Deviation
BMI	26.27 kg/m^2	4.423 kg/m^2
HT	1.607m (1.51m female – 1.697m male)	0.236m
CR	1.006 mg/DI	0.125 mg/DI
AGE	40.6 years	8.717 years
SEX	-	0.48 female – 0.52 male
WT	69.16 kg	22.633 kg
BSA	1.747 m^2	0.4012 m^2
CRCL	96.31 mL/min	34.533

Table S4.3: Correlation matrix of generated covariates

	BMI	HT	CR	AGE	WT	BSA	CRCL
**BMI**	1.00	-0.05	-0.04	-0.02	0.46	0.32	0.43
**HT**	-0.05	1.00	0.01	0.04	0.85	0.93	0.75
**CR**	-0.04	0.01	1.00	-0.01	0.00	0.00	-0.34
**AGE**	-0.02	0.04	-0.01	1.00	0.03	0.03	-0.20

**WT**	0.46	0.85	0.00	0.03	1.00	0.98	0.89
**BSA**	0.32	0.93	0.00	0.03	0.98	1.00	0.88
**CRCL**	0.43	0.75	-0.34	-0.20	0.89	0.88	1.00

Table S4.4: Parameter of the model of the simulated scenario

**Param**	**True Model used for simulation**
CL	0,648
V	2,259
BMI on CL	0,2014
CRCL on CL (exponential)	0,3063
BSA on V	0,0958
SEX on V	-0,3207
Additive Error	0,1
Proportional Error	0,001
CL - inter-individual variability (random effects)	0,2
V - inter-individual variability (random effects)	0,2
Correlation between CL and V (random effects)	0,05

Configuration file for the simulated scenario

model=SimulatedModel.mod

continuous_covariates=BMI,HT,CR,AGE,WT,BSA,CRCL

categorical_covariates=SEX

[test_relations]

CL=BMI,HT,CR,AGE,WT,BSA,CRCL,SEX

V=BMI,HT,CR,AGE,WT,BSA,CRCL,SEX

[valid_states]

continuous=1,2,3,4,5

categorical=1,2
