# Supplementary material for: Go beyond the limits of genetic algorithm in daily covariate selection practice
Source: J Pharmacokinet Pharmacodyn. 2023 Jul 26;51(2):109–21. doi: 10.1007/s10928-023-09875-7 (PMC10982092; doi:10.1007/s10928-023-09875-7)
Supplement: Supplementary file 4 — Supplementary file4 (PDF 94 KB) [file 10928_2023_9875_MOESM4_ESM.pdf]

Journal of Pharmacokinetics and Pharmacodynamics (Original Paper)

TITLE:

Go beyond the limits of Genetic Algorithm in daily covariate selection practice

Authors: D. Ronchi[1], E.M. Tosca[1], R. Bartolucci[1,2], P. Magni[1]

Date: Received: data/ Accepted: date

1. Dipartimento di Ingegneria Industriale e dell'Informazione, Università degli Studi di Pavia, 27100 Pavia, Italy
2. Clinical Pharmacology & Pharmacometrics, Janssen Research & Development, Beerse, Belgium

Corresponding author:

Paolo Magni paolo.magni@unipv.it

Section 5: Additional information for the Remifentanil case study

Configuration file

model=RemifentanilModel.mod

continuous_covariates=AGE,HT,WT,BSA,LBM

categorical_covariates=SEX

[test_relations]

CL=AGE,HT,WT,BSA,LBM,SEX

V1=AGE,HT,WT,BSA,LBM,SEX

Q2=AGE,HT,WT,BSA,LBM,SEX

V2=AGE,HT,WT,BSA,LBM,SEX

Q3=AGE,HT,WT,BSA,LBM,SEX

V3=AGE,HT,WT,BSA,LBM,SEX

[valid_states]

continuous=1,2,3,4,5

categorical=1,2

Table S5.1: Covariate models selected by different runs of GA (First 5 runs)

**Model parameter**	**Covariates**				
	**GA run#1**	**GA run#2**	**GA run#3**	**GA run#4**	**GA run#5**
CL	AGE – exponential<br>WT – exponential	AGE – exponential<br>WT - exponential	AGE – exponential<br>WT - exponential	AGE – exponential<br>WT – exponential	AGE – exponential<br>BSA - linear
V1	LBM - exponential	AGE – power<br>WT – exponential<br>SEX - linear	LBM - exponential	LBM - exponential	BSA - linear
Q2	AGE – exponential	AGE – exponential	AGE – exponential	AGE – exponential	AGE – exponential
V2	AGE – exponential<br>SEX - linear	AGE – exponential<br>SEX - linear	AGE – exponential<br>SEX - linear	AGE – exponential<br>SEX - linear	AGE – exponential<br>SEX - linear

Q3	AGE – hockey stick	AGE – hockey stick	AGE – hockey stick	AGE – hockey stick	AGE – hockey stick
V3	AGE – exponential<br>HT – hockey stick<br>SEX – linear	AGE – exponential<br>SEX - linear	AGE - exponential<br>SEX - linear	AGE – exponential<br>HT – hockey stick	AGE - exponential<br>SEX - linear

Table S5.2: Covariate models selected by different runs of GA (Last 5 runs)

**Model parameter**	**Covariates**				
	**GA run#6**	**GA run#7**	**GA run#8**	**GA run#9**	**GA run#10**
CL	AGE – exponential<br>HT - exponential	AGE – exponential<br>BSA - linear	AGE – exponential<br>HT - exponential	AGE – power<br>HT - exponential	AGE – exponential<br>LBM - exponential
V1	LBM - exponential	BSA - linear	AGE – exponential<br>LBM - exponential	LBM - exponential	LBM - exponential
Q2	AGE – exponential	AGE – exponential	AGE – power<br>BSA - exponential	AGE – linear	AGE – exponential
V2	AGE – exponential<br>SEX - linear	AGE – exponential<br>SEX - linear	AGE – exponential<br>BSA – linear<br>LBM - exponential	AGE – power<br>SEX - linear	AGE – exponential<br>SEX - linear
Q3	AGE – hockey stick	AGE – hockey stick	AGE – hockey stick	AGE – hockey stick	AGE – hockey stick
V3	AGE – exponential<br>SEX – linear	AGE – exponential<br>SEX - linear	AGE - exponential<br>HT – hockey stick	AGE – exponential<br>SEX - linear	AGE – exponential<br>HT – hockey stick<br>LBM - exponential
