# Supplementary material for: Go beyond the limits of genetic algorithm in daily covariate selection practice
Source: J Pharmacokinet Pharmacodyn. 2023 Jul 26;51(2):109–21. doi: 10.1007/s10928-023-09875-7 (PMC10982092; doi:10.1007/s10928-023-09875-7)
Supplement: Supplementary file 5 — Supplementary file5 (PDF 105 KB) [file 10928_2023_9875_MOESM5_ESM.pdf]

Journal of Pharmacokinetics and Pharmacodynamics (Original Paper)

TITLE:

Go beyond the limits of Genetic Algorithm in daily covariate selection practice

Authors: D. Ronchi[1], E.M. Tosca[1], R. Bartolucci[1,2], P. Magni[1]

Date: Received: data/ Accepted: date

1. Dipartimento di Ingegneria Industriale e dell'Informazione, Università degli Studi di Pavia, 27100 Pavia, Italy
2. Clinical Pharmacology & Pharmacometrics, Janssen Research & Development, Beerse, Belgium

Corresponding author:

Paolo Magni paolo.magni@unipv.it

Section 5: Detailed information about the models selected on different simulated dataset.

Green highlights the true covariates introduced into the selected model with the true form, yellow highlights the true covariates introduced with a different form, and red highlights the incorrect covariates introduced.

Table S5.1: Covariate models selected in the 20 different simulated dataset (8 samples/id).

8 samples / id															
**BMICL**	**BMIV**	**HTCL**	**HTV**	**CRCL**	**CRV**	**AGECL**	**AGEV**	**WTCL**	**WTV**	**BSACL**	**BSAV**	**CRCLCL**	**CRCLV**	**SEXCL**	**SEXV**
4	0	0	0	0	0	0	0	0	0	0	4	4	0	0	1
0	0	4	0	0	5	0	0	0	0	3	0	4	4	0	1
4	0	0	0	0	0	5	0	0	0	0	4	4	0	1	1
4	0	0	0	0	0	0	0	0	0	5	0	4	0	0	1
5	0	0	0	0	0	0	0	0	4	0	0	4	0	0	1
4	0	4	0	5	0	0	0	0	0	0	4	0	0	0	1
4	0	4	0	5	0	0	0	0	0	0	5	0	0	0	1
2	0	0	0	0	0	0	0	0	5	0	0	4	0	0	1
2	0	0	0	0	0	0	0	0	0	0	0	3	4	0	1
5	0	0	0	4	0	0	0	4	4	0	0	0	0	0	1
4	0	0	0	0	0	0	0	0	0	0	5	4	0	0	1
4	0	0	4	0	0	0	0	0	0	0	0	4	0	0	1
2	0	0	0	0	0	0	0	0	0	0	2	4	0	0	1
4	0	0	0	0	0	0	0	0	0	0	4	4	0	0	1
2	0	0	0	0	0	0	0	0	5	0	0	4	0	0	1
4	0	0	0	0	0	0	0	0	0	4	0	4	0	0	1
4	0	0	0	5	4	0	0	0	0	0	0	4	4	0	1
4	0	0	0	0	0	0	0	0	0	0	0	3	4	0	1
4	0	0	0	0	0	0	0	0	0	4	4	4	0	0	1
4	0	0	0	0	0	0	0	0	4	0	0	4	0	0	1

Table S5.2: Covariate models selected in the 20 different simulated dataset (5 samples/id)

**5 samples / id**															
**BMICL**	**BMIV**	**HTCL**	**HTV**	**CRCL**	**CRV**	**AGECL**	**AGEV**	**WTCL**	**WTV**	**BSACL**	**BSAV**	**CRCLCL**	**CRCLV**	**SEXCL**	**SEXV**
4	0	0	0	0	0	0	0	0	0	0	4	4	0	0	1
5	0	0	0	0	0	0	0	0	0	0	4	4	0	0	1
4	0	0	0	0	0	0	0	0	0	0	4	4	0	1	1
5	0	0	0	5	0	4	0	0	0	2	0	0	4	0	1
4	0	0	4	0	0	0	0	0	0	0	0	4	0	0	1
2	0	0	0	0	0	0	0	0	0	0	4	4	0	1	1
5	5	0	0	5	0	5	0	0	0	2	0	0	0	0	1
5	0	0	0	0	0	0	0	0	0	0	5	4	0	0	1
5	0	0	0	0	0	0	0	0	0	0	0	3	4	0	1
4	0	0	0	0	0	0	0	0	0	0	0	4	0	0	1
4	0	0	2	0	0	0	0	0	0	0	0	4	0	0	1
4	0	5	0	5	0	4	0	0	0	0	4	0	0	0	1
0	0	4	0	0	0	0	0	0	0	0	0	4	4	0	1
4	0	0	0	0	4	0	0	0	0	0	0	4	0	0	1
5	0	0	0	2	0	0	0	4	4	0	0	0	0	0	1
5	0	0	0	0	0	0	0	0	0	0	0	4	0	0	1
4	0	0	0	0	0	0	0	0	0	0	4	4	0	0	1
4	0	0	0	0	0	0	0	0	0	0	0	3	4	0	1
4	0	0	0	4	0	0	0	4	0	0	4	0	0	0	1
4	0	0	0	0	0	0	0	4	0	0	4	4	0	0	1

Table S5.3: Covariate models selected in the 20 different simulated dataset (3 samples/id)

3 samples / id															
**BMICL**	**BMIV**	**HTCL**	**HTV**	**CRCL**	**CRV**	**AGECL**	**AGEV**	**WTCL**	**WTV**	**BSACL**	**BSAV**	**CRCLCL**	**CRCLV**	**SEXCL**	**SEXV**
5	0	0	0	0	0	0	0	0	0	0	5	4	0	0	0
4	0	0	0	0	0	0	0	0	0	0	4	4	0	1	1
5	0	0	0	0	0	0	0	0	0	0	4	4	0	0	1
4	0	0	0	0	0	0	0	0	0	2	4	4	0	0	1
4	0	0	0	0	0	0	0	0	0	0	4	4	0	0	1
2	5	0	0	0	0	0	0	0	0	0	0	4	0	0	1
2	0	0	0	0	0	0	0	0	0	0	5	4	0	0	1
5	0	0	0	0	0	0	0	0	0	0	4	3	0	0	0
5	0	0	0	0	0	0	0	0	0	0	0	4	4	0	1
5	0	0	2	0	0	0	0	4	0	0	0	4	0	0	1
4	0	0	4	5	0	0	0	0	0	4	0	0	0	0	0
0	0	5	0	0	4	0	0	4	0	0	0	4	4	0	1
4	0	0	0	0	0	0	0	0	0	0	0	4	0	0	1
5	0	0	0	0	0	0	0	0	0	0	2	4	0	0	1
4	4	4	0	4	0	0	0	0	0	0	0	0	0	0	1
4	0	5	5	0	0	0	5	0	0	0	0	0	0	0	1
4	0	0	0	0	0	0	0	0	0	0	0	3	4	0	1
4	0	0	0	5	0	0	0	0	0	4	2	0	0	0	1
4	0	0	0	0	0	0	0	0	0	0	2	4	0	0	1
4	0	0	0	0	0	0	0	0	0	0	4	4	0	0	1
